# Identifying key factors for successful formulation and implementation of healthcare policies on non-communicable diseases: a multinational analysis

**DOI:** 10.3389/fpubh.2024.1292176

**Published:** 2024-02-08

**Authors:** Abdulfatai Olamilekan Babaita, Yasmin Jahan, Ryota Nakamura, Michiko Moriyama

**Affiliations:** ^1^Graduate School of Biomedical and Health Sciences, Hiroshima University, Hiroshima, Japan; ^2^Hitotsubashi Institute for Advanced Study, Hitotsubashi University, Kunitachi, Japan

**Keywords:** health policy, non-communicable disease, prevention, multisector, determinants of health, health equity

## Abstract

**Objectives:**

Non-communicable diseases (NCDs) are a major public health concern that accounts for 74% of global deaths each year. The increasing burden of NCDs exhausts public health resources and threatens the achievement of the 2030 agenda for sustainable development. The purpose of this study is to thematically analyze the contributory factors in the health policy process and reforms to strengthen the prevention of NCDs across borders, as well as the milestones achieved through the process of policy-making, change, and implementation.

**Method:**

This study informs and draws on the findings of contributory factors in the health policy process for preventing NCDs across borders: United States, England, Sweden, Bangladesh, Singapore, South Korea, and Thailand. Ten experts from the seven countries were recruited purposively for a semi-structured interview (e-Interview) on the NCD policy-making process in their countries, either through health ministries or the authors’ network. This descriptive qualitative study design is guided by the “Three I’s” framework of public policy (institutions, ideas, and interests). In addition to the information obtained from the interviewee, data were also sourced from relevant documents and homepages suggested by the interviewee, as well as health homepages of the countries.

**Result:**

The following themes were generated: (1) environmental policies and social determinants, (2) multistakeholder involvement, (3) interministerial collaboration, (4) independent evidence and review institution, (5) integrated health data, and (6) primary care system. There was a shift from individual-targeted policies to environmental policies and social determinants. Notably, national campaigns were developed through non-governmental organizations (NGOs) for the primary prevention of NCDs.

**Conclusion:**

The shift from behavioral modification and treatment to social determinants is important. NCDs are broad and require a multisector and multilevel approach. Establishing an organization or hierarchical body to overlook NCDs could result in increased awareness, focus, and surveillance and enhance the policy process.

## 1 Introduction

Non-communicable diseases (NCDs) are a major public health concern that accounts for 41 million deaths each year; this is an estimated 74% of global deaths compared to 63% in 2008 (1, 2). In comparison to two decades ago, NCDs are causing an increase in mortality than ever. In 2000, 4 out of the top 10 causes of death globally were due to NCDs; currently, it has increased to 7 out of the top 10 causes of death (3). According to World Health Organization (WHO) projections, by 2030, NCDs will account for 55 million deaths if measures are not adequately established (4). The human race is in a battle that shifts from communicable diseases (CDs) to NCDs.

The population is dying prematurely from NCDs (17 million people), especially in low- and middle-income countries (LMICs). The deaths from major NCDs are from four groups of diseases: cardiovascular diseases (CVD), cancers, respiratory diseases, and diabetes (1). There is an interconnection between these diseases in terms of risk factors: physical inactivity, harmful use of alcohol, tobacco use, and unhealthy diets (1). Despite these shared risk factors that could facilitate the smooth process in the prevention and reduction of deaths, the incidence and mortality rate from NCDs still persists.

The key to addressing NCDs is through prevention, screening, detection, and treatment, which require a great deal of active national policies, strong political commitment, functional programs, and collective efforts (1, 4, 5). However, the work and progress in NCD policy formation and implementation is still a notable concern in many countries (6). According to the NCD Countdown 2030 report (6), a collaboration among WHO, Imperial College London, NCD Alliance, and The Lancet of the 186 countries tracked on progress in achieving Sustainable Development Goals (SDGs) target 3.4 of a one-third reduction in NCD mortality by 2030, less than half of countries are on the course to achieve the one-third reduction rate. For the majority or more than half (including the two most populous countries in the world, China and India), the target will not be achieved by 2040, and policy reform is imperative (6).

There is a high prevalence and increased deaths from NCDs in LMICs, and the common trends contributing to the incidence should not be ignored in developed countries. NCDs have a multifactorial etiology, and the influence of socio-economic factors cannot be underestimated. Urbanization is contributing to the diet culture, the propensity for a sedentary lifestyle, and harmful recreational activities, which are linked to the occurrence of NCDs.

The WHO developed the “Global Action Plan” on policies that will impact the reduction target when implemented (4). Several countries have been implementing various policies and measures (international and national) to combat the challenges of NCDs. The purpose of this study is to thematically analyze the contributory factors in the health policy process and reforms to strengthen the prevention of NCDs across borders and the milestones achieved through the process of policy-making, change, and implementation. The findings from this research would help policymakers globally to identify the gaps within the existing policies and introduce the key measures and interventions to control the prevalence of NCDs.

### 1.1 Health sector policy

The WHO defines health policy as “the decisions, plans, and actions (and inactions) undertaken to achieve specific health care goals within a society or undertaken by a set of institutions and organizations, at national, state and local level, to advance the public’s health” (7). To build on this, health policies are not only about contents or white papers but they extend beyond acts enactment. To understand policy and avoid exclusive focus on policy content, which was the direction of early health policy research, a few frameworks have been developed for policy analysis (8, 9).

The “Three I’s” framework posited that the interrelation of institutions and actors’ interests and ideas are the prime directive to analyze health sector policies. The political science model has been extrapolated to the analysis of healthy public policy and impacted the policy process in health issues (10). In healthcare, the framework has been used to aid analysis in studies of evidence-informed policies about health systems, pharmacogenomics policy development, and NCDs policy (11–13). For this study, this framework could illuminate the processes involved from the formulation to the implementation of NCD policies.

### 1.2 The “Three I’s” framework

Due to the complexity of the policy process, understanding policy-related difficulties and situations requires a great deal of consideration of the drivers of policy. The focus of the “Three I’s” framework relatively explains the true nature of policy change.

The institution refers to the context and processes of the political structure. This can be viewed as the rules governing the political process. There are three integral institutional dimensions, which can shape the political structure: policy agency, policy authority, and policy process. Policy agency “refers to ways institutions structure who gets to exercise power.” In the case of policy authority, the point is “what elected officials can do” and the policy process “refers to how decisions are made” (14). Therefore, the distribution of power, who makes decisions, and to what extent power can be exercised are salient in process negotiations, formulation, implementation, and evaluation of NCD policies.

Understanding of the institution is not absolute; the interest and power bestowed on policy actors are significant in the policy process (14). Actors involved in policy have varying interests and agendas. Moreover, the policymakers (in government) are also influenced by interests that may rather lead to inaction in some aspects of policy (15). This explains the lack of interministerial collaboration hindering the multisectoral action plan in NCDs policy, and the various ministries have different overriding concerns. It is not always a win-win for some ministries. The interest of some industries, such as the tobacco and alcohol industries, may not align with the action plan of the health sector, which may lead to conflict and misrepresentation in the policy formation process.

On the other hand, “ideas” are an integral part of understanding policy formation and implementation. Ideas in policy context are the values, preferences, knowledge, and experiences of actors. This shapes the priority of agenda-setting in the policy process (15). The ideas in the institutions will determine the rationality behind some NCD policy processes. Is the institution keen on rationalizing the policy through research evidence, preferences, expert opinions, or some other values? The alignment of values of actors proves protective or disruptive of the NCD policy process (15).

## 2 Method

This study informs and draws on the findings of contributory factors in the health policy process for the prevention of NCDs across borders: United States, England, Sweden, Bangladesh, Singapore, South Korea, and Thailand. This is a descriptive qualitative study design guided by the “Three I’s” framework of public policy (institutions, ideas, and interests).

The researcher requested that the Japanese embassy in high Healthcare Index countries contact the countries’ Ministry of Health/Ministry of Public Health to introduce eligible personnel for the study. It was imperative that many countries participate, but due to other priorities (mainly COVID-19), as cited by countries and participants, the final set of countries was subject to availability. The selection criteria for participation were those from the Ministry of Health, the Ministry of Public Health, and academia (research); those involved in planning and who have an overall understanding of the country’s health policy. There were no restrictions on job title or function. Therefore, the eligible participants were decided by the participating countries’ ministries and subsequent authors’ networks; only the countries with participants who consented were interviewed and included in the study.

A semi-structured interview (E-Interview: Zoom) was adopted to inquire from key informants who are relative players in the policy-making process. A total of 10 participants were interviewed in the study (England:1, United States: 2, Sweden: 1, Bangladesh: 1, Singapore:1, South Korea: 2, Thailand: 2). The participants include government officers (Thailand, Sweden, England, and Bangladesh) and researchers in the relevant fields (United States, Thailand, South Korea, and Singapore). Some participants with academic affiliations were government-appointed in their respective policy authorities. In the case of two participants from one country, they were either interviewed together in one sitting (South Korea) or interviewed independently. In the latter case, the interviews were transcribed independently, and a summary of the findings was presented for final analysis. In addition to the information obtained from the interviewee, data were also sourced from relevant documents and homepages suggested by the interviewee, as well as health homepages of the countries.

The interview guide was developed by the research team based on previous literature (16), and in line with and understanding of the proposed theory of the complex inter-relationship between institutions, ideas, and interests in policy-making. The contents are about the actual policy in terms of the background of NCDs, policy development process, actors and sectors negotiation, implementation, and evaluation context. The interview guide is presented in Table 1.

**Table 1:** Interview guide.

1.What is the main focus of NCDs in your country? Please give us the reasons. (We can check by epidemiological open data)
2.Will you please introduce counter policies against the NCDs? Is there any philosophical background about the policies, or evidence you are based on?
3.What are the primary care measures/frameworks your country has to prevent NCDs? Please introduce them. (When we receive keywords, we can search them on the website or other materials.)
(Do you have any specific or general healthcare policies for the prevention of NCDs?)
4.What is the process of policy formation in your country?
5.What are the factors you consider before any policy formation and implementation in your country?
6.What do you think is the most significant policy intervention that your country has implemented so far?
7.How have you evaluated the NCD measures? Evaluation axis (healthcare expenditure, the prevalence of the diseases, QOL, the well-being of citizens?) and indicators.
8.What are the challenges you face in the process from disease identification to policy formation and in the pre and post-policy implementation phase?
9.How do you think the current global pandemic would affect the progress of NCD prevention in your country as well as globally?

The data collection started in the fiscal year (FY) 2020 and went through FY 2022. The phase of the interview was conducted by an interdisciplinary team of researchers experienced in policy-making and research (3rd author: specialized in health economics and engaged in NCD policy research; 4th author: specialized in chronic care and healthcare research with work experience at the Ministry of Health). During the data collection phase, reflexivity was ensured at the end of each interview session by creating a report and peer debriefing (17). The interview sessions were video recorded for transcription; these were accomplished by verbatim and intelligent transcription. For coding, an individual theme was used as the unit of analysis to capture an idea. In a concept-driven approach, codes were outlined before the coding process; however, to compensate for the bias in this analytic preconception, codes were also sourced in a data-driven approach (18). The interview extracts were manually analyzed for each country by one of the researchers and subsequently combined to generate final themes. The process of analysis was discussed by the research teams, and a consensus was reached on the emerging themes. In addition, the researchers triangulated the findings by sourcing data from relevant documents and governmental homepages. Website content was included in the findings as supplemental; this was so that not every aspect of the inquiry was readily available online or published. There were some convergences of findings, and inconsistencies in published information and interview results were revalidated with the participants. In case of conflicting information in countries with two interviewees, this was member-checked, and consensus was considered for the final result.

## 3 Results

To simplify the themes and connect with the proposed framework, three of the themes generated were related to the healthcare system (focus, integrated health data, and primary healthcare system) and the others were related to the structure of the policy process, which could be explained by the 3 I’s framework. Table 2 describes the summary of the interview.

**Table 2:** Summary of the interview.

Themes	United States	England	Sweden	Thailand	South Korea	Singapore	Bangladesh
NCD Focus	The focus is usually state specific with footprints of “Healthy People 2030.” CVD; Cancers; Diabetes; Respiratory diseases; Mental Health, and some Dementias; Obesity.	i.CVD (ischemic heart disease; stroke) Cancer Diabetes COPD, Mental ill health/ Dementia; ii.Social determinants of health (equity) NCD risk factors	i.Cancer Secondary prevention of stroke Mental health CVD Chronic lung disease Diabetes However, the focuses are system effectiveness and social determinants of health	i.Diabetes; Hypertension. CVD; Cancers; COPD chronic kidney disease (due to its the burden on health expenditure) There is a transition to sociodeterminants of health like tobacco, alcohol, sugar, and trans-fat.	CVD COPD Cancers Diabetes	i.Diabetes: The declaration of “war on diabetes” was a placeholder to represent or address other diseases. This is a red herring that is used to improve physical activity which could have an impact on obesity and improve general well-being; ii.Health Promotion	CVD (especially hypertension) The prevalence of hypertension is steadily increasing; there is collaboration with the USA on Resolve Hypertension Program Diabetes COPD Cancers
Counter Policies	i.Tobacco tax ii.Unhealthy products tax (e.g., salt) iii.Alcohol restriction (varies by state) iv.Social support	i.Obesity plan (including childhood obesity) ii.Salt reduction program/ industry iii.Sugar tax	i.Alcohol sale restriction ii.Prohibition of smoking in public places iii.Monitoring system on drug abuse and gambling	i.Healthy Lifestyle Strategic Plan (2011–2020) ii.Sugar tax iii.Ban of trans fat in domestic and imported food products iv.Alcohol Control Act v.Ban tobacco use in public places	i.Health Plan 2030 ii.Tobacco tax of 70% of the retail price iii.In door places in public transportation are completely smoke-free through iv.Passenger Transport Service Act and Railroad Safety Act v.Healthy Eating: School meal Act was enacted in 1981 on mandatory nutrition standards for schools vi.Cancer screening by NHIS vii.Metabolic screening in Seoul city viii.National health examination strategy	i.Healthy hawkers and healthier choice symbol ii.National Steps Challenge Program iii.Community health assist scheme iv.Social security policy	i.National Tobacco Control Cell (Act 2005): by 2040 tobacco free ii.The work on alcohol reduction, unhealthy diet (salt and trans-fat), and inadequate physical activity are under review
Primary Care Measure	i.Screening and Counseling ii.Community needs assessment through the Affordable Care Act iii.Campaigns	i.General practitioner in the primary setting are the gatekeepers ii.Integrated care from different level of providers	i.The expansion of primary care to be a bigger part of healthcare and involvement in prevention	i.Campaign on physical activity is becoming a social trend; celebrity encouragement of physical activity ii.Health examination survey targeted at effective coverage	i.The primary care system is underdeveloped ii.Mass media and campaigns	i.Prevention programs mostly driven by the Singaporian Health Promotion Board under the ministry of health	i.Screening: Population level ii.Advocacy and Capacity Development: Population level
	iv.The use of “Hotspotting” and “Superutilizers” v.Patient-Centered Medical Home vi.Accountable care organizations vii.Pay for performance compensation viii.The use of wellness applications	iii.Screening and early detection (breast, cervical, bowel screening) iv.HPV vaccination through schools NHS health check at the age of 40 v.Metabolic screening for over age 40 vi.Diabetes program and weight monitoring vii.Social Prescribing viii.Dementia Screening	ii.In the school system, there is an educational program linked to alcohol, narcotic drugs, dopping, and tobacco use which all pupils have to go through. There is free school lunch.	iii.NCDs Health checkup in each primary care center iv.Volunteer village healthworker for screening v.Screening (e.g., liver fluke in northeast region) vi.HPV vaccination for 5th grade school girls vii.Health promotion and risk reduction program	iii.Smoking Cessation: There is smoking cessation treatment that involves initial payment from the client. The reward after successfully completing the program is refund of the payment to the client; it becomes free.	ii.Campaigns and Programs: An example is the Healthy Hawker Food Initiatives and Healthy Choice Logo iii.National Steps Challenge: A physical activity initiative by the HPB iv.National Health Survey for Screening v.Health Behavior Transformation Coaching Program	
Process of Policy Formation		It starts with strategy by independent bodies (PHE) and the department of health to develop a strategy and set up an ambition. In order to set up the policy, there is series of consultations with stakeholders (e.g., patient group), evidence based review, and drawing on the experience of other countries. Then, there is further consultation based on the findings before the development of actual legislation.	Thorough investigations, not by different authorities, but inquiry. The ministry through government can give assignments to the authority (i.e., The Swedish Agency for Health Technology Assessment and Assessment of Social Services). The authority would submit report based on extensive findings which the government could act on.	The policy option is set by analyzing empirical evidence in the sittings of mutistakeholders such as NGOs, members of academia, and national health secretary office to name a few. National Health Commission Office (NHCO) works with every stake holders in the society to discuss a certain significant health related problems. National Health Commission Office (NHCO) is assigned to deputy prime minister.	In Korea, the policy is formed by committee, usually experts, where they suggest a policy, and is becomes policy after the government acceptance. The Typical Process of Policy Formation: 1.Political Pressure in Congress: Debate on Policy Formation 2.Ministry: Evidence Review The Alternative Track: Policy is suggested through research evidences to the government for consideration. The National Evidence-based Healthcare Collaborating Agency (NECA) is for evidence generation.	The determination of priority research area. Then, the interest in policy development comes from the data from expertise, hospitals, healthcare and universities. Sometimes funding is provided to investigate a problem or evidences from countries like US and UK. The impact is calculated before initiation. There is also consultation with doctors and international committee.	Policy Development: Multisectoral Action Plan 1.Preparation of Operational Plan to the Steering Committee. 2.Steering Committee approves, reject, or modifies the Policies and Acts. Planning: Ministry of Health and Family Welfare: The Chief is the Secretary with other Secretaries. Program Management and Monitoring Unit (PMMU): They deal with all kind of policy related to Health.
			Sometimes, topics are discussed on a nonpolitical level to the ministry level, and later escalation to the prime minister. The stakeholders are usually involved in some ways, however, on the political level, the kind of nonpolitical servants are not included in that discussion.	This is further escalated to the cabinet for approval according to policy agenda.			Steering Committee: Members are usually formed from the Health and Non Health Sector; the Directorate General of Health Services (DGHS) and all other Directorates remains in the steering committee.
Key Factors in Policy Formation	i.Medical association ii.Stakeholders lobbying activity iii.Think tank Research Institute iv.Centers for Disease Control and Prevention v.National Institute of Health vi.World Health Organization	i.The process of transparency and consultation. There is also a process called write round where all other sectors and government have opportunity to give feedback if they have any concerns. ii.NICE Imapct assessment: This needs to be done because there are health impacts. Also, other impacts across government are considered. iii.Equity iv.Accountability	i.Expected outcomes ii.Effect on economy iii.Effect or devaluation of public health. iv.If it is changing legislation, it is quite extensive step that proceeds this kind of decision making. If it is a minor issue, the foregoing investigations might be to a small extent.	i.Burden of disease ii.Risk factor iii.Evidence based information iv.Cost effectiveness and resources v.Feasibility vi.Stakeholders opinion	i.Public opinion ii.Evidence based information (NECAHIRA, KDCA, KHPI) iii.Political pressure iv.Specific events	i.Fairness in policy (e.g., equity, ethnicity) ii.Budget impact analysis iii.Cost effectiveness analysis iv.Health burden v.6 yearly health survey	i.Expert opinions ii.Evidences iii.Prevalence of NCDs
Significant Policy Intervention		i.Tobacco control: Adult smoking rates are below 15% and continue to go down. The ambition for smoke free generation. ii.Salt reduction iii.Primary care iv.Universal health coverage v.Independent Evidence and Review Institution	Cancer streamlined treatment procedure	i.Sugar tax ii.Ban of trans fat iii.Alcohol Control Act iv.Tobacco control Act: Ban of tobacco use in public and less pubic advertisement	i.Cancer screening: most of the cancer screening has reduced the prevalence significantly; survival rate has significantly improved through screening ii.The Health Check-Ups: Finding the hypertensives and diabetic has increased the live years. These are determinants of cost effectiveness	i.National Step Challenge ii.Healthy hawkers iii.Active Singapore campaign iv.Strengthening infrastructure (e.g., building parks)	i.Establishment of NCD corner at each primary healthcare center. ii.Building the NCD Screening iii.Work on risk factors (e.g., tobacco)
Key Performance Indicators	i.Cost effectiveness ii.Quality and Outcome Framework (QOF)	i.Cost effectiveness ii.Independent monitoring and accountability system of disease burden and impact iii.Equity gap	Patient outcome is a strong indicator. There is large financial investments on data registries based on quite specific treatment or patient groups.	i.National health survey to check the health status for NCDs ii.Burden of diseases study every 4–5 years. iii.Electronic medical record like HITAP to measure healthcare cost and compliance of treatment. iv.Report on Morbidity and mortality every 2–3 years	i.There is cancer management law and data regarding every cancer is publicly monitored and released: Prevalence, Incidence, Mortality, and 5 years survival. ii.In other diseases, there is before and after policy change assessment (through studies.to determine usefulness of policy; sometimes, there is no study. iii.Health expenditure	There is no feedback for it; the monitoring and evaluation is still in the preliminary stage and there is recognition to improve it.	i.Operational Level Indicators: For OP level of NCD Time wise Year wise Achievements ii.Disbursement-Linked Indicator: Wider variation Provision of at least 2 antihypertensive and 2 antidiabetic drugs throughout the country. Management model in Primary Health Complex iii.SDG Global Indicator: 5 targets concerned with the NCD iv.Health expenditure
Challenges in the Process of Policy Formation	i.Lobbyists ii.Politician perspective of regarding or disregarding science	There is always an opposition and therefore there is consultation and the need to publish the findings.	i.The Politian perspective of regarding or disregarding science	i.Changing key policy makers ii.Insufficient evidence based supporting document	i.The biggest challenge is there is no hierarchical national body (leadership institute.to contact for NCDs; It is fragmented. ii.Interministerial interference: Ministry of finance and health opposing opinion in tobacco tax. iii.Ignorance of parliament members	i.Finance ii.Care effectiveness challenges iii.The work on determinants of health (e.g., multiethnicity, equity) iv.Introduction of sugar taxation	i.Lack of Central Body or Institution on NCD: This can serve the purpose of NCD Directorate.
			ii.The transition of policy implementation from the central to the counties. iii) The transition to public-private partnership. iv) Powerful industries influence	iii.There are some groups of people under enterprise support provoked to act against certain policy or law formulation. iv.Previous cost-effective intervention with relative impact	iv.No equity related research v.No national agenda for NCDs vi.The loss of focus on social determinants vii.NCDs policy are institutional based rather than population based		ii.Politicians, general public and specialist are still focusing on communicable disease which delays action on NCDs policy implementation. iii.Monitoring and Surveillance System: This is the weakest part of the program. iv.Integration of Health and non-health sectors in the decision making v.Functional Implementation rather than policies and guidelines in books vi.Lack of Essential Package: Drugs, Diagnostics (e.g., serum creatinine) vii.Lack of Formal Up-ward and Down-ward Referral between the Community Clinic and Secondary and Tertiary Facility

### 3.1 Environmental policies and social determinants

The key factors in several countries were environmental and social policies. An overriding concern of the policies was to mitigate the environmental and social determinants of health linked with NCDs. All the countries have at least one, if not all, of the following policies tackling NCDs: tobacco tax, alcohol control act, trans fat ban, physical activity initiatives, salt reduction program, and obesity plan. Instead of a solitary policy on tobacco tax, there is a prohibition of smoking in public places to tackle the effects of secondhand smoking.

In South Korea, the Health Plan 2030 (HP2030), which is the 5th national health plan, is a national masterplan established to promote the national policies for health promotion and disease prevention. The overarching goal is “extending healthy life expectancy and promoting health equity” (19). According to the HP2030, relevant policies have been conducted (19, 20). Tobacco tax is 70% of the retail price, and in-door places in public transportation are completely smoke-free through Tobacco Control Act; this includes a policy implemented on attaching graphical health warnings on cigarette packages and banning the display or advertisement of tobacco products in stores. On the other hand, a smoking cessation treatment initiative involves initial payment from the client, and the reward after successfully completing the program is the refund of the payment to the client; it becomes free. The prevalence of tobacco use is high but has decreased compared to two decades ago; a steady decline has been observed throughout the years in men.

In England, the focus is healthy life expectancy; this centers on prevention, early detection, and effective management of a disease (21). There is an improvement in life expectancy, but more years of ill health and a large inequality gap between the rich and poor. The life expectancy gap between the rich and poor is 9 years. However, the healthy life expectancy gap is wider and could be about 19 years. Hence, the target is finding people from an early age or individuals with disabilities due to NCDs and creating a burden for the family and society. To address the determinants of health, there is improvement in housing standards, food quality, and sanitation, to name a few. For example, with the propagation of a healthy lifestyle, smoking rates reduced from 29% in 1990 to less than 15% in 2017 (21). The predominant philosophy in the National Health Service (NHS) is utilitarian, but inequality and equitable approaches are considered. Under the Equality Act of 2010, there is an obligation for policies to consider the potential impact of inequality (22). Moreover, one of the themes of the NHS Long Term Plan (improvement of service, health, and wellbeing), a plan for the next 10 years developed by the experts and patients’ groups, is to prevent illnesses and address health inequalities (23).

In the United States, Healthy People 2030 sets the footprint for the national objective to improve health and wellbeing (24). One of the goals of the national agenda prioritized sociodeterminants of health and states that “create social, physical, and economic environments that promote attaining the full potential for health and well-being for all” (25). In the USA, there was work on urban gardening, a social support project funded by the Centers for Disease Control and Prevention (CDC). Funding was provided to cities as a community activity that enables inhabitants to grow fresh fruits and vegetables. The government is also working toward ensuring equity in the population.

In Singapore, the Health Promotion Board, a statutory body under the Ministry of Health aligned with the government, championed initiatives named “Health Hawker Program” for vendors in food courts and the “Healthy Choice Symbol” for foods, beverages, and meals (26). These initiatives are to promote healthy eating and are part of the public campaign of “War Against Diabetes” (26, 27).

In Thailand, the national strategic plan is the Healthy Lifestyle Strategic Plan (2011–2020) (28). The health promoting hospitals engage with the communities and local governments at the village level for community-based intervention to build the enabling environment, reduce NCD-risk in the community, and enhance health literacy in the community. For environmental determinants, tobacco is banned in public places with less public advertisement. Prevalence of current tobacco use in persons aged 15+ years decreased from 21.4% in 2011 to 19.1% in 2017.

### 3.2 Multistakeholder involvement

The involvement of multistakeholders in the process of policy formation has broadened the perspectives and improved the compliance of individuals and industries to policy changes regarding NCDs; stakeholders are used as agents of change in society. The stakeholders include, but are not limited to, academia, healthcare professionals, industries, patient groups, health organizations, non-governmental organizations, and non-profit organizations.

In Thailand, the National Health Commission Office (NHCO) engages every stakeholder in society to discuss a certain significant health-related problem; the NHCO is assigned to deputy prime minister. The Thai Health Promotion Foundation (THPF) is like a social lubricant, and there is a law on allocating a certain proportion of tax on alcohol and tobacco to the THPF. At the academic research level, there is work with lawyer, academics, and finance to go beyond public health to tackle the problem. Health Non-Governmental Organizations (NGOs) receive funds from Thai Health to work against sugar consumption; starting from the dentist group, they discuss with the public, and it creates trust. By law, government officers are allowed to work with health NGOs and foundations. There is a lot of trust in charitable organizations and health NGOs, which helps them function smoothly. NGO’s work is not only from the public but inclusive of doctors; there are Health NGO’s emergency doctors fighting against alcohol abuse. Moreover, monks are funded to convince people not to consume alcohol for at least 3 months for religious reasons. Some monks became champions for the Thai Health Promotion Foundation; they are motivated to be involved with people by privately visiting them at home. Religious leaders (i.e., monks) are empowered to be a part of ministry, establish public forums, and be involved with academics, ministers, politicians, and players to challenge industries. Collaboration with industries is not a huge success; a lot happens behind public platforms.

In 2012, Public Health England (PHE) was established as a Care Act and involves teams working on alcohol, obesity, and tobacco, including technical teams in the Department of Health and Social Care. The establishment of PHE has played a major role in translating generated evidence for policymakers (29). The PHE oversees and publishes guidelines on the reduction and reformulation program, which includes the sugar and salt reduction program (30). Similarly, the Food Standards Agency enforces and ensures the safety and hygiene of food (31). In each iteration of a voluntary food reformulation program, there is engagement with industries and civil society to ensure program feasibility; there are several consultations with the target set by the government agencies (30). With the engagement, industry compliance was achieved. Based on the program, there is independent monitoring of the salt content of food to set the targets; sodium urinary studies have been conducted periodically every 3–4 years to determine the impact of consumption. In the PHE report on the 2017 salt reduction target, 80% of the overall products for in-home and 70% of the overall products for out-of-home consumption achieved the maximum target set (32). Correspondingly, there is progress in the reduction target on the sugar contents of foods and drinks (30). To keep the policy-making evidence focused and unbiased by different stakeholders, the engagement is managed with a clear distinction between the industry being a stakeholder for consultation and not being involved in the policy-making decision. In terms of the tobacco industry, England scores low on the Global Tobacco Industry Interference Index (GTI: 32), the best for the countries in the study (33).

In Singapore, the government is keen to involve industries in developing healthier solutions. In contrast to the approach in many European countries, Singapore’s government helps and even subsidizes, the development of new products, such as high-fiber noodles, with food and beverage manufacturers.

### 3.3 Interministerial collaboration

The findings from this study reveal that the collaboration of several ministries in implementing some NCD-related policies are considered by the interviewees to be important. Conflicts can occur when the ministries’ policy goals do not align (34). Such policies include tobacco tax, sugar tax, salt intake, and alcohol restriction.

In Thailand, the key player is the Ministry of Public Health, which administers alcohol and tobacco policy. However, on taxation policy, the Ministry of Public Health needs the support of the Ministry of Finance and the Ministry of Commerce on request for minimum price. Moreover, THPF supports other ministries, including the Ministry of Transport, to ensure more public transport; it was realized that public transport makes people physically active. There is also the Ministry of Tourism and Sports supporting running events and the Ministry of Natural Resources and Environment boosting city parks to promote physical activity.

In Singapore, government involvement in health is broader than the Ministry of Health alone and integrates other ministries. The Ministry of Health collaborates with the Ministry of Environment on public health, such as vector control; they work with the trade industries on tobacco, taxation, and food importation, and with the Ministry of Manpower on occupational health.

In Sweden, the interest among different ministries may differ with respect to the prevention and control of NCDs. There is a process to arbitrate conflicted interests among ministries to facilitate collaboration, in which the prime minister decides how the ministries act in different aspects. At first, discussions are on a non-political level, and if agreement is not reached due to different opinions, the question is escalated to the state secretaries. Then, if consensus is not achieved, the prime minister presides. On the political stage, the non-political servants are not included in the discussion.

### 3.4 Independent evidence and review institution

Independent evidence and review institution could be key and denote financially and organizationally autonomous from the government authority and other stakeholders with vested interests. Few of the study countries have independent evidence reviews and institutions, including the use of international bodies’ guidelines or evidence, including WHO and OECD. Notably, according to the interviewees, two of the countries have drawn on the experience of other countries in the implementation of NCD prevention programs or treatments (25).

In England, the establishment of PHE in 2012 as a Health Act provided more freedom to work independently in generating/publication of evidence; they have good relationships with academia and other research institutes. This serves as a bridge between finding evidence and implementing it. There are independent Scientific Advisory Committees (SAC) providing evidence-based support and challenging the scientific evidence used in policy development; they work with the scientific committees of the Department of Environment, Food, and Rural Affairs (Defra) (35). On the other hand, England draws on the evidence of international organizations such as WHO and emulates other counties’ policy after practical analysis and usefulness of the policy. The country is looking to Scotland, which introduced a minimum unit price for alcohol, to thoroughly understand the benefits on health before implementation.

In Sweden, there is an independent national authority named The Swedish Agency for Health Technology Assessment and Assessment of Social Services (SBU). The organization is entrusted with collating evidence from peer-reviewed papers or articles in different fields; they synthesize evidence on different implementations related to health and social care. The evidence is summarized to help the policymakers in government and committees that produce health and treatment guidelines (36). It is a small agency but works extensively regarding the collection of evidence-based policy-making. Moreover, the ministries, through the government decree, can assign responsibility to the authority. The authority can report their findings to the government; the government cannot influence the outcome. Although they work through the government decree, they owe the government no loyalty. Moreover, the WHO and the Organization for Economic Co-operation and Development (OECD) are influential for recommendations in healthcare.

In South Korea, the National Evidence-based Healthcare Collaborating Agency (37) (NECA) is for evidence generation; Health Insurance and Review Assessment (38) (HIRA) is an administrative organization. The NECA is 100% funded by the government; however, it functions independently to provide evidence for policy. Moreover, international recommendations influence policy-making in Korea; the WHO recommends the implementation in most Korean ministries. The WHO Framework Convention on Tobacco Control (FCTC) influenced the enforcement of smoking cessation and tobacco-related policies.

### 3.5 Integrated health data

The availability of integrated electronic medical records in some countries contributes to successful surveillance and continuity of care. The patients’ information is essential in improving policy and assuring population-based health planning.

In Sweden, there is a National Patient Register (NPR) featuring inpatient and outpatient healthcare; it ensures the integration of patients’ healthcare data in a single database. This is maintained by the Swedish National Board of Health and Welfare (39).

In England, NHS Digital, also known as the Health and Social Care Information Center, is the national body that collects, protects, and shares patient information. NHS Digital manages a central system through the patients’ records shared by various health and care organizations. The data are used in policy formation, health and care improvement, and betterment of service delivery (40).

### 3.6 Primary healthcare system

The existence of a primary healthcare system in the prevention of NCDs was evident in the countries. The study countries’ primary healthcare system targets mitigating some risk factors, if not all. A series of initiatives are implemented in primary care, such as screening and detection, monitoring and surveillance, community needs assessment, education in increasing health literacy and disease management, treatment, and follow-up to reduce morbidity and mortality.

In Thailand, the primary care measures to prevent NCDs are implemented by health-promoting hospitals (each health center covers a population of 3,000–5,000 people and has a team of 3–5 nurses and paramedics) and the communities. Apart from basic curative services, the primary care measures for NCDs include general health promotion, risk assessment (diabetes/hypertension/cervical cancers/depression/dementia screening), and specific NCD risk-reduction programs. At the village level, there are about 10 village health volunteers each village who provide health information/education to the community and support health center staff in conducting community-based diabetes/hypertension risk screening and other activities. The health centers also work with the communities and local governments at the village level for community-based intervention to build an enabling environment, reduce NCD risk in the community, and enhance health literacy. Thailand has a basic health benefit package of the Thai Universal Coverage scheme for general healthcare policy; renal dialysis is part of the health benefit package. In addition, there are some specific healthcare policies for NCD prevention in Thailand, for example, human papillomavirus (HPV) vaccination for 5th-grade school girl and screening for liver fluke in the north-east region to prevent Cholangiocarcinoma. To address the risk factors, there are the Alcohol Control Act, Tobacco Control Act, sugar control, and physical activity strategies. Health examination surveys as a part of effective coverage in terms of screening are better compared to a decade ago. The coverage has moved from prevalence-oriented to outcome measures; the focus is to measure the level of control for the treatment of hypertension, diabetes, cervical cancer, etc. However, it is a challenge to gain the compliance of patients.

In England, the health service has realized the key to reducing burden is prevention. General Practitioners (GPs) in the primary setting are the gatekeepers; the patients’ initial visit is with the GP, and they can be referred to specialists in complex cases. This prevents the exhaustion of secondary and tertiary care, makes the system more efficient, and reserves the funds. There is screening, early detection, and diagnosis for which patients do not have to necessarily visit a GP; the nurses can do the screening in primary care settings. For cancers, the program has long been established, for example, cervical screening, which is conducted in primary care by nurses. There is HPV vaccination through schools on the community level; breast and bowel screening is part of public health. The health system has moved to institute regional plans with community and outcome measures; this creates the incentives for the health services to prioritize improving health outcomes of people rather than the number of services. For cardiovascular diseases and diabetes, there is a joint practice between general practice and public health in the local government program. The National Health Service (NHS) health check is targeted at people at the age of 40 by checking blood pressure, cholesterol, and blood sugar for advice and information. Moreover, they provide dementia screening, information on healthy lifestyles, and prevention of cardiovascular diseases (41). The facilities are available in general practice and other settings; patients are rereferred from general practice to GP for preventive measures. For example, for change in diet and exercise, patients are followed up for 6 months, and even if no changes are seen, the follow-up care is in general practice.

In the United States, the Affordable Care Act mandated health systems to conduct community needs assessments to recognize community needs; the Act changed the landscape of the health system. That was a real paradigm shift because, previously, hospitals took care of people without follow-up. There is a dynamic system, for example, the Centers for Disease Control and Prevention (CDC), which is part of the National Institute of Health; the CDC has a National Center for Chronic Disease Prevention and Health Promotion. There are incentives built into various types of programs such as Accountable Care Organization (ACO) and Medicare Advantage to prevent NCDs. Prevention has become an integral part of primary care; it is one of its main tenets. In primary care, there is a “huge void” of physicians due to physician’s preference for specialty care; however, other practitioners, such as nurses and physician assistants, are built into the system to provide and support primary care. The form of reimbursement is still a big issue in primary care; there is a relative value unit. Physicians are accountable for their time based on the number of patients they see. Hence, the time is not enough to provide counseling around prevention. Therefore, many places are working toward more integrated models of care with different levels of providers caring for a patient: the social worker, a nurse practitioner, nutritionist, and mental health provider. The physicians can provide medical care, and other team members take care of social determinants of health and health behavior issues. The primary care model is evolving, and this evolution is using the levers of reimbursement to incentivize higher-quality primary care, including prevention. To reach the uninsured, there are federally qualified health centers that are the healthcare safety net for the uninsured. However, they are typically under-resourced and operate on a low budget; there may be a long waiting time and difficulty in getting an appointment. There are campaigns for health screening, but they are not mandated. The system is transitioning from incentivizing volume to incentivizing value. Similarly, Jeff Brenard developed programs such as “hotspotting” and “superutilizers” to address the sociodeterminants of health and their large impact on health and costs. The use of wellness applications is also introduced by some insurers.

In Sweden, primary care is much smaller than specialized care. There is work in progress on making primary care a bigger part of healthcare. They are more involved in the care of people in society. Preventive care mainly occurs in primary care for the first doctor visit, although it can also be in specialized care. Throughout the system, commercials are not commonly used for prevention; it is driven within the healthcare system (primary or specialized care). In addition, the public health agency provides some life factors concerning alcohol consumption, drug use, etc. As part of primary care, there is an educational program linked to alcohol, narcotic drugs, doping, and tobacco use for school pupils. The full length of prevention is by the counties, in some instances, by the municipalities.

Bangladesh is a limited resource country, including healthcare professionals. To cover the gap, the primary care system is used as the first-line point for NCD measures; there are community clinics and primary health complex in the system. Each community clinic is run by the Community Healthcare Provider (CHCP) and Health Assistants; they are equipped with blood glucose strips and a digital sphygmomanometer. To achieve more manpower and stable workforce, people from the community are trained on a specialized course to be utilized in the system. There is a national protocol for referral to the primary health complex. In the primary health complex, there are outdoor and indoor facilities; NCD corner is established in each primary health complex; they have a trained nurse, sub-assistant community medical officer (SACMO), and a doctor. According to the government, they should be provided at least two antihypertensive drugs and one diabetes drug; three antihypertensive drugs and two diabetes drugs are available across the primary health complex in the country for free. The drug is provided for at least 1 month before a recheck, and patients are provided an NCD book. However, constant drug supply is a big issue. According to a pilot in the third sector program (2011–2016) by the Ministry of Health and Family Welfare (MOHFW) and Government of Bangladesh (GOB), a 5-year program, there is a total of 430 primary health complexes and the system is currently in 70 health complexes (42). The target is to complete 200 health complexes in the fourth sector (2017–2022). The focus is to complete all the Primary Health Complex in the next sector, from screening to follow-up. There is a plan to develop an NCD management model, formal referral to secondary or tertiary facilities, and provision of drugs at the community clinic due to proximity.

### 3.7 Challenges

There are several major specific and common challenges in the study countries. In most countries, politicians’ perspective of formulating policy is a major challenge hindering the progress of NCD policies. Another challenge is overcoming the lobbying activities of the industries. On the other hand, opposing evidence and lack of evidence to support the choice of policy are an impediment to the policy process. Finance is not a major challenge except for Bangladesh; however, in all the countries, the priority and funding of intervention is an impediment in the negotiation process. Moreover, the difficulty in establishing a central body or institution for NCDs to oversee the affairs has received little relevance and consideration. It is recommended that there is a need for a high-level leadership institute in charge of NCDs.

In Bangladesh, the lack of a central body that serves the purpose of the NCD directorate is one of the challenges. Similarly, there is a weak surveillance system on risk factors. In addition, there is a lack of essential packages such as drugs and diagnostics in primary care. Moreover, there is also a lack of formal upward and downward referral between the community, secondary, and tertiary facilities.

In South Korea, the biggest challenge is that there is no national body to contact for NCDs. Similarly, there is no national agenda on NCDs; it is healthcare or disease category specific. Furthermore, interministerial interference, where there is opposing opinion on tobacco tax between the ministry of finance and the ministry of health, is a challenge.

In Sweden, on different levels of the government, the politician’s perspective drives or hinders policy formation. The transition between policy formation by the central government and implementation at the county or municipality level might be an issue because of the different political leadership. In addition, as noted by the interviewee, there can be “different interest groups which might influence the political leadership on the kind of low level, which might disturb the ambitions from the central government idea of policy formation. This decentralization might apparently be a problem for the central government.”

In Thailand, in the policy formulation phase, policy approval may be delayed due to changes in the responsible person; especially policy that involves law and legislation. Similarly, there are insufficient evidence-based supporting documents during the window of opportunity for policy formulation. Moreover, the lack of cooperative participation among multi-partners is a challenge in the post-policy implementation phase. NCDs are associated with people’s behavior, and the policy never fits all. In some instances, the inability of some past interventions to achieve the expected cost-effectiveness is also a challenge for future action.

## 4 Discussion

The NCDs focus in the study countries is in terms of the risk factors and the four major diseases, in addition to dementia and mental health. There has been a shift and recognition by the institutional structure to focus more on NCDs, except for Bangladesh, which is struggling with the prioritization of NCDs over CDs. On the other hand, Thailand adopted nine national strategies and a national NCD strategic plan to address the effect of NCDs on the health budget for Universal Health Coverage (UHC); 74% of deaths in Thailand are due to NCDs from the four major diseases (43). Although the priorities have been determined based on the prevalence and incidence of the diseases in the countries, there is the same pattern of disease focus, such as diabetes, cancers, COPD, and CVDs, as these account for the major burden of NCDs globally (1).

Instead of focusing solely on diseases, policies were formulated to address the determinants of ill health. This comes from the perspective of preventing NCDs as it is less expensive, and due to the multifactorial nature (1, 44), the work on the determinants of health is different in the countries. In terms of ratification of the WHO Framework Convention on Tobacco Control (FCTC) by the countries, only the USA has not ratified (45). The treaty was signed by the Secretary of Health and Human Services when it was open for signature, but it was never ratified by a two-thirds vote in the Senate. Although, in 2009, the USA passed the Family Smoking and Prevention Control Act (FSPCA), which comprises many of the FCTC elements and permits the USA Food and Drug Administration (FDA) to regulate the tobacco industry, its implementation is still challenged by the industry (46). This may be due to the strong influence of the tobacco industry as the USA scores higher on the GTI (33) (GTI: 76) than other countries included in the study.

Meanwhile, the primary care system was established as the safety net for the prevention of NCDs in some of the study countries by using the system to reach and educate the people in the community. The system ensures easy access to screening, treatment, and follow-up. The components and structure of the primary care system could serve as the guiding principle in addressing NCDs. It is argued that the use of primary care is more grounded when consolidated with public policies regarding risk factors (47). In terms of the implementation of some activities and initiatives regarding the prevention of NCDs, this can best be addressed on the primary level of prevention in the primary care system; health promotion and disease prevention are cores in primary care (48). In addition, primary care is one way to address the social determinants of health (48, 49), and one of the objectives of the global action plan with UHC as an overarching principle for the prevention and control of NCDs (49). The WHO noted that “for universal health coverage (UHC) to be truly universal, a shift is needed from health systems designed around diseases and institutions toward health systems designed for people, with people” (49).

Interministerial collaboration is acknowledged by the interviewees as important in NCD prevention and control. It is not necessarily well-documented in the study countries because the effectiveness of such collaboration on the policy impacts is hard to quantify. This is also reflected in recent debate about the challenges and opportunities of such collaboration in policy implementation (12, 34, 50). Internationally, the degree to which a collaboration is achievable depends on countries and specific policy contexts. Our analysis of the interview data highlighted some (at least partial) success in collaboration between ministries in Thailand for implementing population-level public health interventions such as alcohol tax. In England, the revenue of the sugar tax has been earmarked to be spent on public health policies; such an arrangement requires coordination between ministries. Ideas agreement, rather than conflict of interest, proves additive and complements the successful implementation of health policies and mitigation of NCD risk factors. Establishing a high-level governance system or process to facilitate interministerial collaborations could be important. Sweden has a process in which the prime minister plays a key role in arbitrating different policy goals between ministries when conflicts arise. It is reported that policymakers in different ministries of Nordic countries cooperate routinely, though achieving policy goals through such collaborations may still pose a challenge (50).

Evidence review and institutions foster evidenced-based decisions in health policies. The value of evidence, rather than trial and error, cannot be overemphasized in the health policy-making process. There is a need to incorporate high-quality research in NCD policies or interventions (51). In this study, independent evidence review and institution were generated as protective against the institution’s biases and influence of actors’ interests. This allows freedom for deliberate review of evidence to guide the policy-making process.

In the study countries, there is a difference in stakeholders and actors’ involvement during the consultation, formulation, and implementation of the policies. In most instances from the countries, stakeholders such as NGOs, industries, and academia are involved; however, the scope of involvement and degree or power of influence vary. The WHO (4) recommended a multisectoral approach to the prevention and control of NCDs. Stakeholders appear as risk and protective factors for rational formulation and implementation of policy. The NGOs are instrumental stakeholders in advocacy and empowerment (52). In Thailand, they are used extensively and engaged in the prevention and control of NCDs. Although the industries are one of the stakeholders that can produce compliance in successful NCD policy, the power, degree of influence, interest, and networks can severe the decision-making process. In Singapore, there are often good relationships between the government and industry when interests align, which can be seen in policies to encourage and reward the creation of healthier food options. It is noteworthy that the tobacco industry still uses indirect strategies such as sponsorship “behind the scenes” to influence and circumvent the smoke-free legislation in Singapore (53), and the influence of industries in some other countries is lobbying, a strategy used by the drink (54) and tobacco industries to influence policies on the governmental level (55). Some studies have highlighted the industries’ (tobacco, alcohol, and sugary drinks) influence as one of the challenges in the policy process (56, 57). The interviewee from the UK noted that the distinction between the industry being a stakeholder for consultation and not being involved in the policy-making decision is important. In support of this, the WHO in FCTC suggests that stakeholders should ensure health policies are free “from commercial and other vested interests of the tobacco industry” (58).

The major challenge common to most countries is the politicians’ perspective regarding the formulation of policy, and this ranges from instability in the political structure, ignorance of policymakers, and disregard for science. The argument is based on priority, funding, and the essentials of implementing a specific intervention. The WHO Independent High-Level Commission of NCDs affirms that a lack of political will, commitment, capacity, and action hinders the implementation of recommended interventions (59). The industries’ interference through lobbying is an obstacle. This is not uncommon as the interest to protect the product and maximize profit will be the utmost priority of the manufacturers despite evidence proving how deleterious the products are. The interference in health policy by the tobacco, alcohol, and sugary drink industries is well documented in some studies (5, 56, 57). Moreso, the tobacco industries are known to misuse and undermine scientific evidence, misrepresent the industries’ contribution to the economy, and fund political campaigns (55). Sometimes, the availability of research evidence to support the intervention is daunting and interferes with the time taken to process a policy. This may be because adopting an intervention is not as important as adapting it to the local context. The process of applying a policy is not only by finding an effective intervention but also by piloting it to ascertain its usefulness in the local context (51). On the other hand, the lack of a leadership institute to guide the direction of prevention and control work on NCD is problematic. Although there are centers for disease control and prevention in some countries, in many countries, the focus is usually adrift to CDs rather than NCDs.

### 4.1 Limitations

In this study, we interviewed one or two informants from each study country. The policy is huge, and the emphasis from each participant may not encompass the depth and multiple aspects of the policies. Therefore, to address this issue, we corroborated and supplemented the findings. In addition, the action plans in each country would best be represented by experiencing the setting and engaging the experts. We endeavored to include more countries to explore varying dimensions of policy and action plans; however, probably due to COVID-19 pandemic, we were unable to receive considerable informants’ consent.

## 5 Conclusion

In this study, we analyzed the health policy process and key factors in successful policy development and implementation of NCDs across borders. The findings suggested the establishment of an organization or hierarchical body to overlook NCDs; NCDs are multifactorial, and the need for a leadership institute as constitutional authority, with ultimate decision, is imperative. This is a recommendation that could result in increased awareness, focus, and surveillance and enhance the policy process as the affair is entrusted to a body rather than being under the general health care system, which contributes to inaction and fragmented responsibility. In terms of policy-making, the shift from behavioral modification and treatment to social determinants is important. NCD is broad and requires a multisector and multilevel approach. The government can set regulations or taxes based on the countries’ value system; in addition, there is a need for population awareness. To achieve this, there is the use of social media, wellness applications, primary care networks, and school health and occupational health through communication of healthcare workers with the community.

The consideration of health equity is key as there is a strategy such as UHC, which ensures low-income people have access to healthcare in poor-budget and poor resource-setting countries; this is also important in high resource countries as there is an economic and gender gap. The review of high-quality evidence during the policy process is a key step in implementing an effective policy or intervention; hence, data resource and integrated health data are crucial and can improve the evaluation, reformation, and improvement of policy. An independent evidenced review and institution can be entrusted with the review, evaluation, and informing the government during policy-making. Further research is required and recommended to narrow down the focus to better understand and provide a full picture of the policy process and action plans.

## Data availability statement

The original contributions presented in the study are included in the article/supplementary material, further inquiries can be directed to the corresponding author.

## Ethics statement

This study was approved by the epidemiological ethics committee of Hiroshima University, Japan. Written informed consent was obtained from the participants for the publication of any potentially identifiable images or data in this article.

## Author contributions

AB: Formal analysis, Conceptualization, Data curation, Methodology, Resources, Validation, Writing – original draft, Writing – review & editing. YJ: Investigation, Resources, Validation, Writing – original draft, Writing – review & editing. RN: Conceptualization, Data curation, Formal analysis, Funding acquisition, Investigation, Methodology, Project administration, Supervision, Writing – original draft, Writing – review & editing. MM: Conceptualization, Data curation, Formal analysis, Investigation, Methodology, Project administration, Supervision, Writing – original draft, Writing – review & editing.
